# Drug Discovery and Development Targeting Dementia

**DOI:** 10.3390/ph16020151

**Published:** 2023-01-19

**Authors:** Agnieszka Zagórska, Anna Czopek, Monika Fryc, Anna Jaromin, Ben J. Boyd

**Affiliations:** 1 Department of Medicinal Chemistry, Jagiellonian University Medical College, Medyczna 9, 30-688 Kraków, Poland; 2 Department of Lipids and Liposomes, Faculty of Biotechnology, University of Wrocław, Joliot-Curie 14a, 50-383 Wrocław, Poland; 3 Department of Pharmacy, University of Copenhagen, Universitetsparken 2, 2100 Copenhagen, Denmark; 4 Drug Delivery Disposition and Dynamics, Monash Institute of Pharmaceutical Sciences, Monash University (Parkville Campus), 381 Royal Parade, Parkville, VIC 3052, Australia

**Keywords:** dementia, drug discovery and development, nanocarriers

## Abstract

Dementia, most often associated with neurodegenerative diseases, affects millions of people worldwide, predominantly the elderly. Unfortunately, no treatment is still available. Therefore, there is an urgent need to address this situation. This review presents the state of the art of drug discovery and developments in targeting dementia. Several approaches are discussed, such as drug repurposing, the use of small molecules, and phosphodiesterase inhibitors. Furthermore, the review also provides insights into clinical trials of these molecules. Emphasis has been placed on small molecules and multi-target-directed ligands, as well as disease-modifying therapies. Finally, attention is drawn to the possibilities of applications of nanotechnology in managing dementia.

## 1. Introduction

Dementia is characterized by cognitive decline involving memory and other domains, including personality, praxis, abstract thinking, language, executive functioning, complex attention, and social and visuospatial skills [1]. Cognitive impairment usually accompanies, and precedes, decreased control over emotional and social responses, behavior, and motivation. More than 55 million people live with dementia worldwide, and nearly 10 million new cases arise yearly [2]. Moreover, as the proportion of older people in the population worldwide is increasing, this number is expected to rise to 78 million by 2030 and to 139 million by 2050 [2]. “Dementia” is also described as a clinical syndrome with different types of dementia, chronic, or progressive brain diseases. Deficits in several cognitive domains, characteristic of dementia, significantly reduce patients’ independence in activities of daily living (ADL). According to the Global Burden of Disease Study 2019, the growth in the number of individuals living with dementia underscores the need for public health planning efforts and policies to address the needs of this group [3]. Moreover, the unmet medical need and relentless growth in dementia cases require extensive efforts by various scientific groups to develop effective dementia treatment and management options. Thus, this review focuses on current strategies and perspectives in drug discovery and development toward dementia.

## 2. Etiology and Pathophysiology

**Figure 1:**
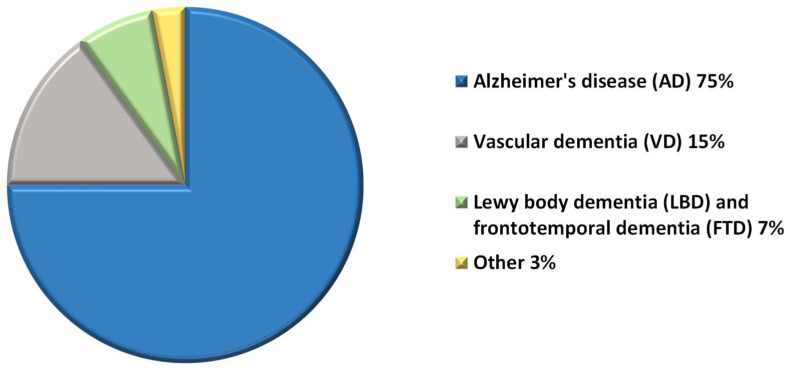
Graphical representation of types of dementia.

Abnormal brain changes cause disorders grouped under the general term “dementia”, but the pathophysiology of dementia is not understood completely. Dementia is most often associated with neurodegenerative diseases, such as Alzheimer’s disease (AD), the most common cause of dementia, and accounts for about 75% of cases [4]. Other causes of dementia include Lewy body dementia (LBD) and frontotemporal dementia (FTD), which contribute from 5% to 10% of all dementia patients [5,6] (Figure 1). However, dementia may also develop in the course of Parkinson’s disease, Creutzfeldt-Jakob disease, and after a stroke, or in the context of certain infections, such as HIV [6,7]. Harmful use of alcohol, repetitive physical injuries to the brain or nutritional deficiencies can cause vascular dementia (VD). Additional risk factors for VD also include hypercholesteremia, diabetes mellitus, hypertension, and smoking, which induce ischemic injury to the brain, leading to permanent neuronal death [7].

**Figure 2:**
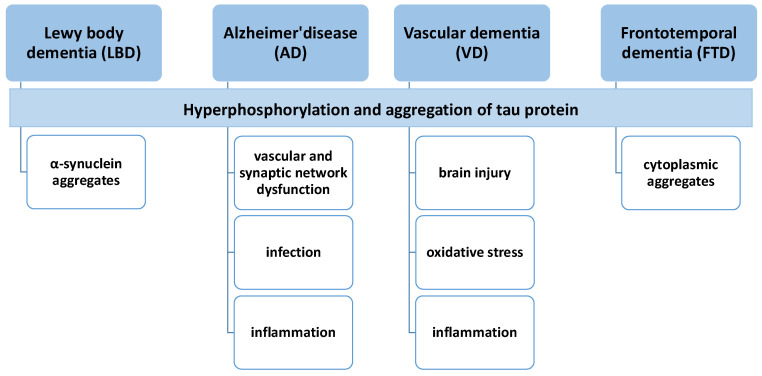
The similarities and differences between the types of dementia, based on their possible pathogenesis.

The pathophysiology of dementia is not understood entirely, but most types of dementia (except VD) are caused by the accumulation of native proteins in the brain (Figure 2).

The pathophysiology of AD has not been definitively elucidated. The amyloid dogma hypothesis, primarily derived from the study of genetic variants of AD, is related to amyloid metabolism. Based on this hypothesis, AD is caused by the accumulation of amyloid in the brain, primarily in its toxic amyloid-β42 (Aβ42) form, which can result in synaptic toxicity. Pathologically, the extracellular Aβ1–42 amyloid aggregates (senile plaques) are formed by proteolytic cleavage of the amyloid precursor protein (APP) with β-secretase, followed by γ-secretase. Neurofibrillary tangles (NFTs), present intracellularly, contain abnormally modified forms of tau protein within affected neurons. While tau proteins stabilize the cytoskeletal microtubules physiologically [8,9], the hyperphosphorylated tau fibrils inhibit axonal transport and lead to cell death. AD dementia is a tauopathy characterized by abnormal hyperphosphorylation of microtubule-associated protein tau that leads to the formation of neurofibrillary tangles. There are also various descriptive hypotheses regarding the causes of sporadic and genetic AD, including the mitochondrial cascade hypothesis [9,10], calcium homeostasis hypothesis [10], inflammatory hypothesis [11,12,13], neurovascular hypothesis [12], metal ion hypothesis [14,15], and lymphatic system hypothesis [14].

In the past two decades, there has been a significant increase in dementia research. The enormous amount of genetic, neuropathological, experimental, and pre-symptomatic biomarker data gathered by independent laboratories suggested that Aβ accumulation is a possible contributing factor to the development of AD symptoms. However, there are some discrepancies between preclinical and clinical results. Lastly, the concern that outcomes reported to date on aducanumab, the humanized IgG1 monoclonal antibody that selectively binds to amyloid β fibrils and soluble oligomers, raised the AD pathology beyond amyloid [15,16,17]. Selkoe noted that the conclusion from the aducanumab case is that “the amyloid hypothesis that we have been working on diligently for over 30 years is scientifically incorrect” [17]. Moreover, manuscripts published in the respected scientific magazine Science contained adulterated images in a series of very influential papers on amyloid and AD [18,19]. All data suggest that the association of amyloid with AD is collateral, rather than causal [15], and there are inconsistencies in the core amyloid hypothesis. Last, but not least, to avoid failures in clinical trials it is essential to make a precise diagnosis of AD/dementia and validate fluid biomarker assays, and not to include patients with unknown amyloid pathologies. With the failure of the amyloid approach, emerging data on the role(s) of vascular, mitochondrial, and synaptic network dysfunction, infection, diabetes, sleep, hearing loss, the gut microbiome, and neuroinflammation/innate immune function are driving research on AD treatment in new directions [15].

Lewy body dementia (LBD) is a neurodegenerative disorder characterized by the accumulation of aggregated forms of the α-synuclein protein in both neuronal and non-neuronal cells in the brain. For these reasons, LBD is classified among α-synucleinopathies, with Lewy bodies or Lewy body diseases (LBDs), which are insoluble aggregates of α-synuclein [19]. Lewy bodies and Lewy neurites produce and accumulate abnormal α-synuclein, which is phosphorylated, nitrated, and truncated, has abnormal solubility, prompts the production of oligomeric species, aggregates into fibrils, and is ubiquitinated [20,21]. α-Synuclein contributes to the fibrilization of β-amyloid and tau, however β-amyloid deposition in the form of diffuse and senile plaques, as well as early changes of neurofibrillary tangle pathology, distinguish LBD from the hallmarks of AD [21,22,23]. Moreover, the symptoms of LBD are more neuropsychiatric, such as dementia-related psychosis (hallucinations and delusions), and patients experience more severe impairment and a more rapid decline in visuospatial function [22,23].

Frontotemporal dementia (FTD) is characterized by the presence of misfolded and dysfunctional proteins aggregated in the grey and white matter of the brain. According to the aggregates’ main protein component, four subtypes of FTD have been named [23]. First, FTD-tau is characterized by hyper-phosphorylated tau protein self-assembled into insoluble filaments [24]. Second are the cytoplasmic aggregates containing abnormally cleaved, hyperphosphorylated, and ubiquitinated and the transactive response DNA 43 kDa binding protein (TDP-43) fragments, which are characteristic features of FTD-TDP-43 [25]. Third, FTD-fused in sarcoma (FUS) FUS-positive, tau/TDP-43-negative inclusions were found in 10% of patients [26]. Lastly, 1% of patients represent FTD-UPS (Ubiquitin/Proteasome System), where the primary inclusions component is unknown but shows immunoreactivity for ubiquitin [27]. However, approximately 40% of individuals with FTD genetic mutations were found to be associated with a specific subtype of aggregate [28,29,30,31]. Regardless of pathogenesis, the hyperphosphorylation of tau results in its disengagement from the microtubules. It induces conformational changes, forming helical and straight filaments of abnormal tau, which aggregate [29]. As in AD, the mechanism of tau hyperphosphorylation remains unknown.

VD can result from cerebral ischemia or hemorrhagic tissue injury in a particular brain region. The heterogeneity of the pathology and clinical features of VD is dependent on the mechanism of the stroke, the degree of tissue loss, and its impact on the connectivity of neural pathways. Due to these factors, a consensus on the defined pathogenesis of VD has not been established. Instead, several mechanisms of brain injury have been proposed, such as cerebral ischemia, glutamatergic and oxidative stress, calcium overload of neuronal cells, immune dysfunction, accumulation of amyloid peptide, the cholinergic hypothesis, and tau proteins [30]. However, on a molecular level, increasing attention is now being paid to inflammation, which contributes to arteriosclerosis and cerebral small vessel disease, which causes cerebral infarction. Emerging concepts in VD were the subject of a comprehensive review by Bir [7].

## 3. Treatment of Dementia

Currently, available treatment options for dementia only have symptomatic effects, and no drugs with disease-modifying properties are available.

The US Food and Drug Administration (FDA) has approved cholinesterase inhibitors and memantine to improve cognitive functions (Table 1). Cholinesterase inhibitors (donepezil, galantamine, and rivastigmine) prevent the breakdown of acetylcholine and can slow or delay the worsening of symptoms. Memantine is an N-methyl-D-aspartate (NMDA) receptor antagonist and decreases the activity of glutamine. Donepezil is approved for all stages of AD, galantamine and rivastigmine for mild-to-moderate stage, and memantine for moderate-to-severe stage disease. However, the efficacy of those approved drugs, based on meta-analysis, is marginal. Thus, cholinesterase inhibitors have a modest beneficial impact on neuropsychiatric and functional outcomes for patients with AD [31]. Memantine, in turn, has minimal efficacy on AD symptomatology, and its safety profile is similar to that of placebo [32]. The clinical benefits of these drugs are related to a delay in the progression of symptoms over several months but have an inconsistent impact on everyday function [33]. Similarly, randomized clinical trials (RCTs) with cholinesterase inhibitors and non-cholinergic drugs in VD reported weak or modest (though sometimes statistically significant) clinical efficacy [34].

Despite the similarity of symptoms, LBD cannot be treated as simply a redux of AD. Thus, there is no FDA-approved drug to address LBD. However, several drugs have demonstrated the potential to serve as disease-modifying treatments for LBD. The most advanced are phase II randomized, double-blind, placebo-controlled studies to evaluate the impact of nilotinib on safety, tolerability, pharmacokinetics, pharmacodynamics, and clinical outcomes in patients with LBD [35]. In recent years, monoclonal antibodies against α-synuclein have been developed. Active and passive immunotherapies against α-synuclein have reduced α-synuclein pathology and associated deficits in rodent models [36]. Moreover, vaccination is increasingly being investigated as a potential treatment for synucleinopathies [37]. Results of the clinical trial on the safety and efficacy of BIIB054, a human-derived monoclonal antibody that targets and binds to α-synuclein, showed that further investigation is warranted to verify the potential of this particular antibody [38,39].

**Figure 3:**
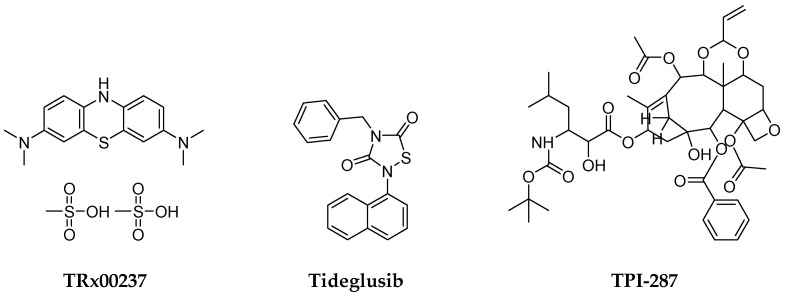
The structures of selective drug candidates for FTD treatment.

Similarly, to date, there are no approved interventional drugs for treating FTD. It is postulated that the prevention of protein misfolding and aggregation, or the clearance of the aggregates, can be the main objectives of therapeutic approaches. Unfortunately, the phase III clinical trial results on a derivative of methylene blue, compound TRx00237, did not suggest benefits in cognitive and motor assessments (Figure 3). Similarly, tideglusib as a glycogen synthase kinase-3 (GSK-3β) inhibitor was ineffective in clinical trials (Figure 3). Some clinical trials focus on recovering loss of function using promotors of microtubule stability, such as davunetide, or TPI-287 (Figure 3). Regrettably, for both drug candidates, no clinical efficacy was observed [39,40].

Implementing medications for dementia-associated symptoms is another important aspect of treatment. Neuropsychiatric symptoms (NPS), also known as behavioral and psychological symptoms of dementia (BPSD), occur in 90% of subjects with dementia. NPS include agitation, aberrant motor behavior, anxiety, irritability, depression, apathy, disinhibition, delusions, hallucinations, and sleep or appetite changes [40]. NPS pathophysiology is usually multifactorial, and contributing factors could be biological, psychological, social, or environmental [41]. Non-pharmacological approaches are recommended as the first choice in the treatment of NPS. Here, it is worth pointing out that there are currently no approved medications for the treatment of NPS in dementia. However, psychotropic medications such as antipsychotics, benzodiazepines, anticonvulsants, and antidepressants are used off-label to treat these symptoms [42,43].

**Figure 4:**
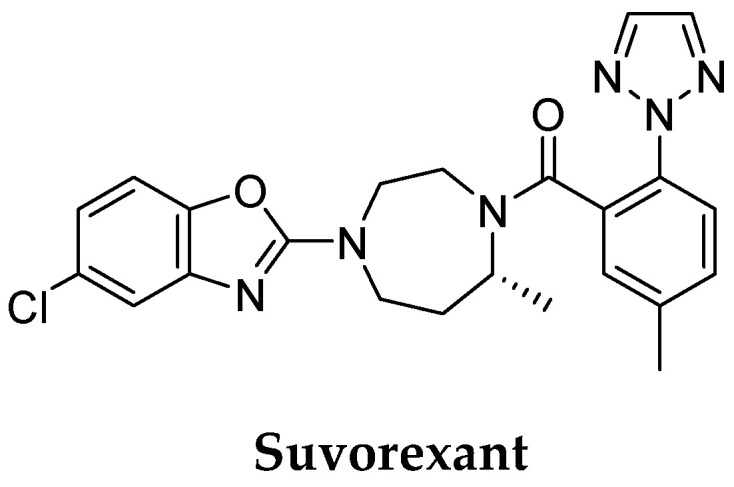
The structure of suvorexant—FDA-approved drug for treating insomnia in patients with mild-to-moderate AD dementia.

At this time, the FDA has approved drugs to address insomnia in people with dementia [43]. The orexin receptor antagonist, suvorexant (Figure 4), is indicated for treating insomnia in patients with mild-to-moderate probable AD dementia. Orexins, also known as hypocretins, are two neuropeptides secreted from orexin-containing neurons, mainly in the lateral hypothalamus. Orexins bind and activate two G-protein–coupled receptors (GPCRs), orexin receptor type 1 (OX1R) and type 2 (OX2R), which activate different downstream signal pathways other than the classical signaling pathways [44]. Moreover, much evidence indicates the role of orexins in human cognition and their close relationship with AD [45].

## 4. Drug Discovery and Development for Dementia

Currently, processes of drug discovery and development (DDD) for dementia address primarily cognitive impairment and neuropsychiatric symptoms of dementia. The strategy of DDD includes drug repurposing, small molecules and multi-target-directed ligands (MTDLs) or disease-modifying therapies.

### 4.1. Drug Repurposing

**Figure 5:**
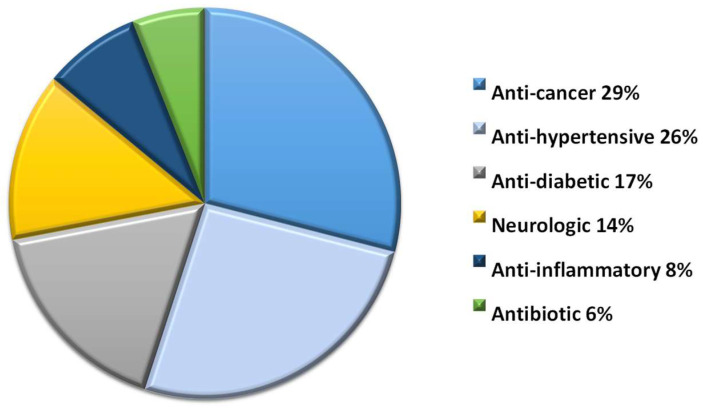
Classification of drugs considered for repurposing in AD treatment.

Repurposing ‘old’ drugs is increasingly becoming attractive as this approach uses de-risked compounds with potentially lower overall development costs. However, identifying drug candidates that can be repurposed for the treatment of dementia needs a lot of effort through data-driven and experimental approaches. They represent six pharmacological groups and represent anti-cancer (29%), anti-hypertensive (26%), anti-diabetic (17%), neurologic (14%), anti-inflammatory (8%), and antibiotic (6%) drugs (Figure 5).

Drugs considered for repurposing in dementia obtained from ClinicalTrials.gov target tyrosine kinases, retinoid X receptors, glucagon-like peptide 1 (GLP-1), peroxisome proliferator-activated receptor-γ (PPARγ), calcium channel, angiotensin receptors and phosphodiesterases (Table 2).

Identifying druggable targets for AD treatment is possible, e.g., by translating genome-wide association studies (GWAS) and multi-omics data using an integrated, network-based artificial intelligence methodology. Clinical data results indicate at least three subgroups of Alzheimer’s patients, each demonstrating a different trajectory of disease progression [46]. Fang et al. identified three drugs (pioglitazone, febuxostat, and atenolol) that are significantly associated with decreased risk of AD [47].

Various anti-cancer agents, such as natural products, nuclear receptors, tyrosine kinase inhibitors, and cytokine modulators, may mitigate AD neuropathology by targeting unique pathogenic mechanisms in AD. Insulin administration could stabilize or improve cognition and memory in the human brain. Thus, AD therapeutics include drugs capable of influencing insulin release (peroxisome proliferator-activated receptor γ (PPARγ) agonists and glucagon-like peptide 1 (GLP-1) analogs). AD and hypertension pathophysiologies are associated with common vascular risk factors, such as vascular impairment and cerebrovascular diseases. However, there are inconsistent results on the impact of controlling blood pressure on the risk of dementia [48]. Even though several FDA-approved drugs have been shown to target risk genes in AD, a detailed understanding of their mechanisms of action and optimal dosage regimes are lacking.

Similarly, drug candidates able to be repurposed for the treatment of LBD were identified in the group of GLP-1 analogs (liraglutide), angiotensin receptor blockers (candesartan), monoamine oxidase inhibitors (rasagiline), and tyrosine kinase inhibitors (nilotinib, bosutinib). Nilotinib is a tyrosine kinase inhibitor that disintegrates α-synuclein and hyper-phosphorylated tau (p-tau) in a mouse model of Parkinson’s Disease [49]. Nilotinib is approved by the FDA as a well-tolerated drug for chronic myelogenous leukemia (CML).

In the last decade, phosphodiesterase (PDE) inhibitors have been of great interest because central nervous system signaling pathways can be influenced by increasing cAMP and/or cGMP. Therefore, PDE inhibitors may have neuroprotective properties and can improve neuronal plasticity in various neurodegenerative diseases [50]. Phosphodiesterase-4 (PDE4) enzymes may be one of the potential therapeutic targets, especially in people with AD who do not have an amyloid burden [51]. The well-known PDE4 inhibitor is rolipram, which crosses the blood–brain barrier and appears most active in the brain [52]. Several studies suggest that PDE4B mediates the beneficial effects of rolipram in animal models of AD. PDE4B is the only subtype of PDE4 expressed in the locus coeruleus, a brain region affected by neurofibrillary degeneration in the early development of AD [53]. In microglial cells exposed to β amyloid, PDE4B transcription is upregulated, resulting in increased TNFα production, but rolipram significantly reduced TNFα release [54]. Moreover, when rats injected with aggregated β amyloid into the hippocampus were subsequently treated with rolipram several days before the training exercise, disturbances in the Morris water maze and passive avoidance tests were reversed [55,56].

**Figure 6:**
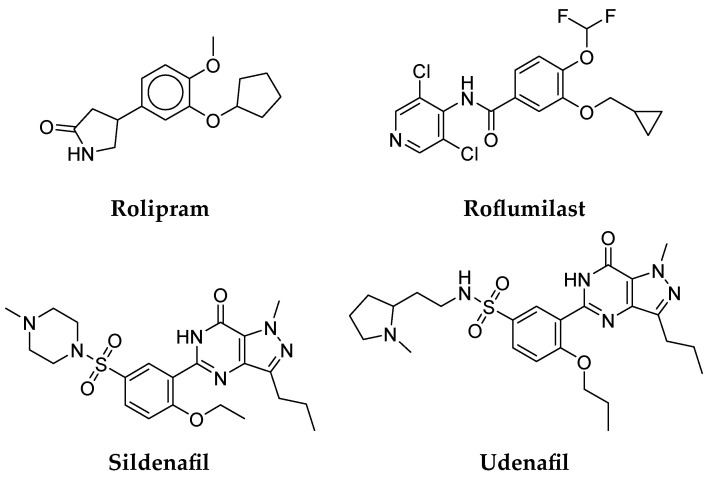
Repurposed PDE inhibitors.

Roflumilast (Figure 6), a potent, selective, and long-acting PDE4 inhibitor, was introduced in 2011 as an anti-inflammatory drug for the treatment of severe chronic obstructive pulmonary disease (COPD). It exhibited fewer emetic effects than rolipram and, although both drugs improved memory in the object localization test, only roflumilast was effective in the spatial Y-maze test. In addition, the combination of donepezil and roflumilast, which, when administered alone, did not improve memory, restored scopolamine-induced object recognition memory deficits in rats [57]. Two randomized, double-blind, placebo-controlled phase I trials were designed to test whether scopolamine-induced cognitive impairment or impairment associated with schizophrenia could be attenuated by the addition of roflumilast (ClinicalTrials.gov ID: NCT02051335, NCT02079844). So far, the results of clinical trials are inconclusive because the combination of roflumilast and donepezil, which was expected to reverse scopolamine-induced cognitive impairment in healthy adults, did not bring significant benefits in humans. At the same time, verbal memory was significantly improved in the participants taking the lowest dose of roflumilast (100 µg) and an antipsychotic drug. Therefore, roflumilast can be suggested as a new strategy for the treatment of cognitive impairments in schizophrenia [58]. Further phase II clinical trials (ClinicalTrials.gov ID: NCT01433666) were conducted to verify whether memory, attention, and information processing improved when the PDE4 inhibitor roflumilast was administered to healthy subjects. The results revealed that the PDE4 inhibitor, roflumilast, had a favourable side effect profile and was effective at the lowest dose of 100 µg [59].

Sildenafil (Figure 6) is the first PDE5 inhibitor marketed for erectile dysfunction and pulmonary arterial hypertension. PDE5 inhibitors increase the level of cGMP, which is lowered in AD. Therefore, they are promising targets in AD. Moreover, cGMP is responsible for the increase in the activity of the peroxisome proliferator-activated receptor-γ coactivator 1α (PGC1α), the overexpression of which suppresses β secretase 1, thus limiting the formation of amyloid-β in AD. Growing evidence indicates that a low dose of sildenafil, via the PGC1α signalling pathway, may suppress not only β secretase 1 expression but also upregulate antioxidant enzymes and increase brain perfusion and neurogenesis, while it suppresses neural apoptosis and inflammation [60]. Despite the cognitive-enhancing effects of PDE5 inhibitors in animal studies, these effects have not yet been translated into clinical trials. To date, sildenafil has been tested in several studies, confirming the improvement of cognitive functions in healthy volunteers [60,61,62]. In these studies, sildenafil administered orally at a dose of 50 or 100 mg only significantly improved mean reaction time in a simple-choice test but did not affect short-term memory or the other psychomotor tasks observed. On the other hand, after a single 50 mg dose of sildenafil, spontaneous neural activity in the hippocampus was significantly reduced, and this parameter was abnormally increased in AD patients. Moreover, the same single dose of sildenafil (50 mg) significantly increased cerebral blood flow and decreased cerebral vascular reactivity. Inconsistencies in the results may be because the study groups were small (6–10 volunteers), the doses used may not be optimal, or multiple dosing may be required to achieve a therapeutic effect. Moreover, the cognitive potential of sildenafil was also tested in patients with schizophrenia and Parkinson’s disease. In these studies, sildenafil did not improve cognitive functions, or the study was discontinued, due to insufficient participation [63,64]. However, a recent study of sildenafil in over 7 million patients found that patients who took this PDE5 inhibitor were 69% less likely to develop AD within six years [65]. Shim et al. reported that chronic treatment with a different PDE5 inhibitor, udenafil (Figure 6), at a dose of 100 mg administered for two months, improved cognitive and frontal executive function [66]. In a subsequent study, a lower dose of udenafil (50 mg) administered for two months also had a cognitive-enhancing effect [66].

### 4.2. Small Molecules

Drug discovery and development pipelines for AD have focused on selective, one-target small molecules for years. The majority of new small compounds act as cholinergic inhibitors (e.g., huperzine A, (−)-phenserine, and ladostigil), decreasing Aβ production by the inhibition of the beta-site APP-cleaving enzyme 1 (BACE1) (e.g., LY2811376, MK-8931, umibecestat, E2609, and JNJ-54861911) or the inhibition and modulation of γ-secretase (e.g., semagacestat, avagacestat, PF-3084014, and CHF5074). Small molecules can also prevent Aβ aggregation (e.g., tramiprosate, scyllo-inositol, epigallo-catechin-3-gallate, and azeliragon) or inhibit tau aggregation. Small molecules that entered clinical trials primarily for AD treatment slow down the progression of the disease and alleviate symptoms but cannot treat the disease. However, with the lack of clear hypotheses regarding the causes of dementia, current experimental studies mainly focus on the major pathogenesis factors and pathways involving numerous receptors and enzymes. Moreover, many biological targets and signaling pathways are involved in the pathology of dementia. There is substantial literature that focuses on drug targets for AD [67,68,69,70,71,72,73], LBD [74,75,76,77], FTD [78,79,80,81], and VD [82,83,84]. As a result of a better understanding of the biochemical regulation of the mechanisms leading to dementia, new molecular targets have been introduced into clinical trials (Table 3).

**Figure 7:**
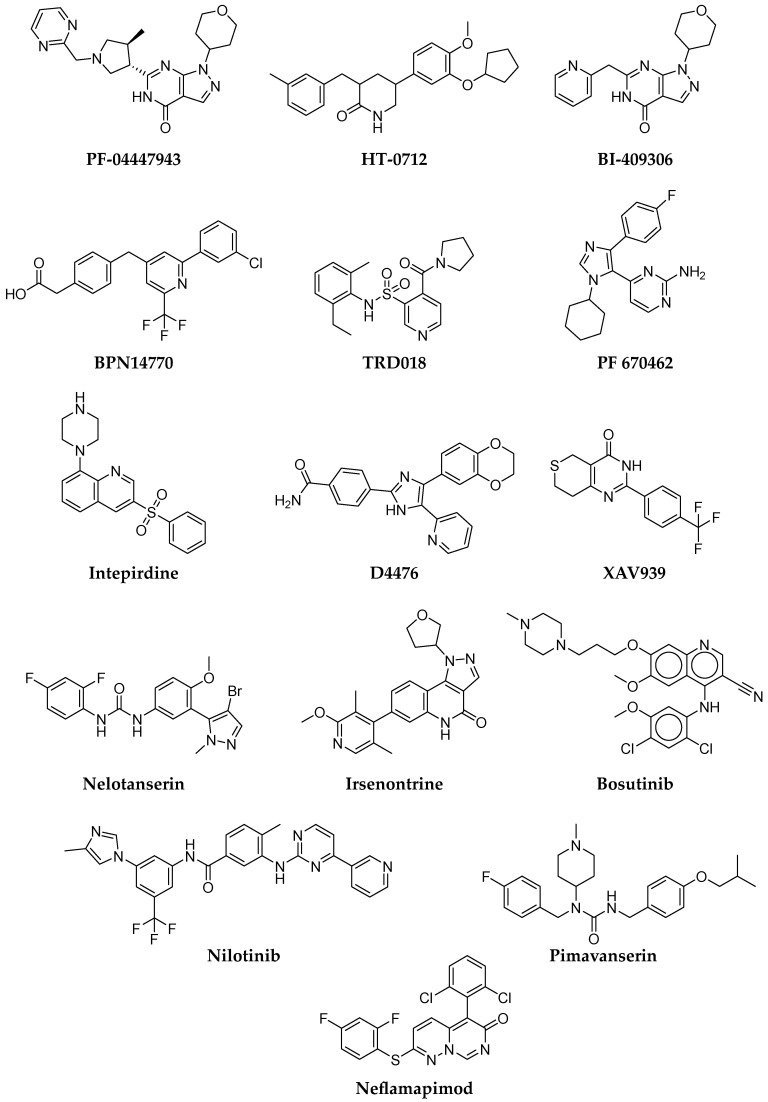
Structures of small molecule drug targets for AD, LBD, FTD, and VD.

There have been several candidate small molecule compounds in development that target PDE4 inhibition. Dart Neuroscience has evaluated a new potent PDE4 inhibitor, HT-0712 (Figure 7). In preclinical studies, HT-0712 enhanced long-term memory formation in young and old mice after a single injection [85]. A recent randomized, double-blind, placebo-controlled phase II clinical trial evaluated the effectiveness of HT-0712 in improving memory and cognitive performance in participants with age-associated memory impairment (ClinicalTrials.gov ID: NCT02013310) [86]. Another PDE4 inhibitor, BPN14770 (Figure 7), developed by Tetra Discovery Partners, has been tested in several phase I and II clinical trials for cognitive impairment and early AD. BPN14770 is a negative allosteric PDE4D inhibitor, which should have fewer adverse effects, including emetic ones. Two randomized, double-blind, placebo-controlled phase I clinical trials (ClinicalTrials.gov ID: NCT02648672, NCT03030105, NCT02840279) investigated single and multiple doses in young and middle-aged healthy participants. In addition to reversing scopolamine-induced cognitive impairment, these studies also assessed the safety, tolerability, and pharmacokinetic profile of BPN14770 [87].

Likewise, a number of PDE9 inhibitors have also been investigated in the clinic. Recently, Pfizer developed a potent, selective phosphodiesterase 9A inhibitor PF-04447943 (Figure 7) that increases cGMP levels in the brain. Phase I clinical trials (ClinicalTrials.gov ID: NCT00988598) assessed the safety of PF-04447943 or placebo, in combination with donepezil, in patients with mild-to-moderate AD [88], while, in phase II clinical trials (ClinicalTrials.gov ID: NCT00930059), its tolerability and effect on the cognitive and behavioral symptoms of AD were examined [89]. Although PF-04447943 was safe and generally well-tolerated, it did not significantly improve cognitive and behavioral symptoms of AD in phase II, compared to the placebo. A new PDE9 inhibitor, BI 409306 (Figure 7), was developed by Boehringer Ingelheim, whose systemic safety in patients with schizophrenia, AD, and healthy volunteers was tested in phase I clinical trials (ClinicalTrials.gov ID: NCT02392468). The randomized, double-blind study showed that the molecule was well-tolerated [90]. However, a double-blind, randomized, placebo-controlled phase II (ClinicalTrials.gov ID: NCT02337907) clinical trial in patients with cognitive impairment due to AD did not show the efficacy of BI 409306 in improving cognitive function in patients with mild AD [91].

Small molecule discovery for FTD was designed to target the modulation of TDP-43-RNA interactions. The small molecule functions by preventing TDP-43 binding to RNA, which may reduce neuronal toxicity. The small molecule, TRD018 (Figure 7), was discovered using computational molecular docking and can displace (G_4_C_2_)_4_ RNA from TDP-43 with an IC_50_ of ~150 μM. Albeit very weak, the compound is the first reported small molecule that prevents the binding of RNA or DNA to TDP-43 [92]. However, Brown et al. postulated that further elucidation is needed to prove that interaction between TDP-43 and RNA or DNA may provide therapeutic benefits [93].

The following drug targets for FTD are kinase inhibitors affecting TDP-43 toxicity and pathology. By way of example, inhibition of casein kinases 1ε (CK1ε) with small molecule inhibitors (PF 670462 and D4476, Figure 7) was shown to reduce TDP-43 phosphorylation [94]. Moreover, poly(ADP-ribose) polymerases (PARP) inhibitors may also provide a therapeutic approach for FTD, through the down-regulation of PAR, to protect against TDP-43 toxicity. An example of this is XAV939 (Figure 7), a potent inhibitor of tankyrase 2 (PARP5B) and a weaker inhibitor of tankyrase-1 (PARP5A), PARP1, and PARP2, which reduced the accumulation of TDP-43 [95].

In past clinical trials for LBD, the long-term safety and tolerability of intepirdine (RVT-101, Figure 7), a selective 5-HT_6_ receptor antagonist, was evaluated. The study was terminated, since intepirdine did not meet primary efficacy endpoints in the lead-in study (NCT02586909). Similarly, both nelotanserin (5-HT_2A_ inverse agonist, Figure 7), and ramelteon (a selective agonist of melatonin receptors MT_1_ and MT_2_), had to be discontinued because of failure to meet efficacy endpoints. Currently, tyrosine kinase inhibitors such as bosutinib and nilotinib are in clinical trials. Bosutinib (SKI-606, Figure 7), a dual inhibitor of the tyrosine kinases (TKs) Abl/Src (approved to treat the chronic phase of Philadelphia chromosome-positive CML), reduces misfolded proteins and protects dopaminergic neurons in animal models. The safety, target engagement, and biomarker effects of bosutinib in LBD showed that the compound, in low doses (100 mg), reduced CSF alpha-synuclein and dopamine catabolism [96]. These results will adequately guide powered studies to determine the efficacy of a dose range of bosutinib and establish a more prolonged treatment in LBD. Nilotinib (Figure 7) is also an Abl tyrosine kinase inhibitor approved for the treatment of CML, which facilitates autophagic clearance of α-synuclein [97]. In a small, open-label, proof-of-concept study, LBD data suggested that nilotinib is relatively safe in patients and has a beneficial effect on motor and cognitive outcomes [98]. A phase 2 RCT to confirm these findings is currently recruiting participants (NCT04002674).

Currently, irsenontrine (E2027, Figure 7), an orally active and selective PDE9 inhibitor, is being studied to evaluate its efficacy, safety, and tolerability in participants with LBD (NCT03467152). Next, pimavanserin (Figure 7), an atypical antipsychotic as a selective inverse agonist of the 5-HT_2A_ receptor, was approved by the FDA in 2016 to treat delusions and hallucinations from psychosis associated with Parkinson’s Diseases. Thus, treating this mechanism may produce benefits, especially in dementias with psychotic symptoms. A phase 3 trial of pimavanserin for the relapse of dementia-related psychosis is ongoing (ClinicalTrials.gov ID: NCT03325556). A phase 2 trial has recently commenced of neflamapimod in LBD (ClinicalTrials.gov ID: NCT04001517). Neflamapimod (VX-745, Figure 7) is a selective inhibitor of the p38 mitogen-activated protein (MAP) kinase alpha enzyme. P38 MAP alpha kinase is an intracellular enzyme involved in inflammation and Aβ-induced and age-related synaptic dysfunction, which may be a driver of learning and memory deficits. The previous study of neflamapimod in AD concluded that the agent might improve episodic memory and might potentially improve amyloid plaque burden [99].

### 4.3. Multi-Target-Directed Ligands (MTDLs)

**Figure 8:**
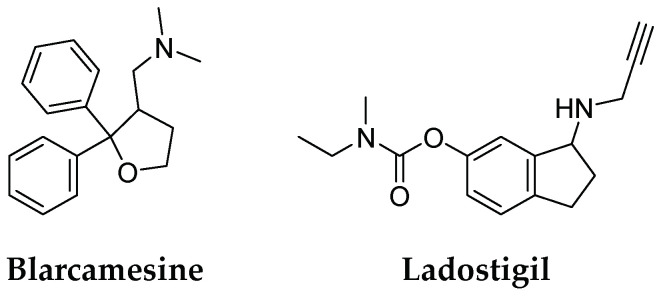
Structures of multi-target-directed ligand targets for AD.

The complex nature of AD and other dementias has led to the development of a multi-target approach. Multi-Target-Directed Ligands (MTDLs) can simultaneously modulate multiple biological targets. For example, compounds that selectively or non-selectively inhibit cholinesterase may help manage AD symptoms based on the “cholinergic theory.” MTDLs based on tacrine, a classical pharmacophore that inhibits both AChE and BuChE at a micro-molar scale, led to the success of memoquin and ferulic acid-memoquin hybrids, which can inhibit both AChE- and self-induced Aβ aggregation [73]. Most novel molecules possess heterodimeric structures designed by combining different pharmacophores, such as existing therapeutics (tacrine, donepezil, galantamine, and memantine), or those derived from natural products [100,101,102,103]. Even though good preclinical results have been reported, there is not a single MTDL in clinical trials. The drug candidates for AD treatment with multimodal action were blarcamesine (ANAVEX2-73) and ladostigil (TV3326) (Figure 8). Blarcamesine acts as a muscarinic receptor and a moderate sigma1 receptor agonist. Lastly, blarcamesine, in a phase 3 trial (NCT04304482), met primary and secondary efficacy endpoints in Rett syndrome [104]. Ladostigil is a dual cholinesterase (ChE) and brain-selective monoamine oxidase-A (MAO-A) and monoamine oxidase-B (MAO-B) inhibitor, indicated for the treatment of dementia, comorbid with extrapyramidal disorders and depression. The compound is based on the combination of carbamate rivastigmine and a N-propargyl scaffold of an anti-Parkinsonian drug and irreversible selective MAO-B inhibitor, rasagiline. In clinical trials (NCT01429623), ladostigil was safe and well-tolerated but did not delay the progression to dementia. Its association with reduced loss of brain and hippocampus volume suggests a potential effect on atrophy [105]. The study also provided Class II evidence that, for patients with mild cognitive impairment (MCI) and medial temporal lobe atrophy, ladostigil did not significantly decrease the risk of the development of AD.

### 4.4. Disease-Modifying Therapies

Drug discovery and development for dementia has shifted to disease-modifying therapies (DMTs). DMTs, including immunotherapy, stem cells, and gene therapy are aimed at preventing, slowing, or facilitating the production and aggregation of pathological proteins. In animal models, both passive and active immunotherapy targeting α-synuclein reduced pathological and behavioral deficits [106,107]. The fully human anti-amyloid monoclonal antibody, aducanumab, received accelerated approval for AD from the FDA in June 2021. It is the first disease-modifying drug for AD, which reduces beta-amyloid plaques and cognitive and functional decline in people with early Alzheimer’s [108]. Aducanumab is classified as a human immunoglobulin gamma 1 (IgG1) monoclonal antibody, crossing the blood-brain barrier and selectively targeting and binding aggregated soluble oligomers and insoluble fibril conformations of Aβ plaques in the brain [109]. Based on weak monovalent affinity, fast binding kinetics, and strong avidity for epitope-rich aggregates, aducanumab has been shown to discriminate between Aβ monomers and oligomeric or fibrillar aggregates [110]. The continued use and full approval aducanumab will require further verification, despite its initial approval being controversial.

Antibodies to α-synuclein have been shown to prevent pathogenic protein spread and promote clearance of aggregates in animal models. Thus, immunotherapies in randomized controlled trials, such as Affitope PD01A and PD03A, target aggregated α-synuclein. PD01A is an active vaccine for α-synuclein. The immunogen is an eight amino acid peptide that mimics an epitope in the C-terminal region of human α-synuclein but with a different amino acid sequence. The vaccine is designed to stimulate B-cell antibody responses but bypass auto-reactive T-cell mobilization, which can elicit harmful neuroinflammatory responses. A phase 1 randomized trial of α-synuclein immunotherapies PD01A and PD03A in multiple system atrophy (MSA) showed that both vaccines were safe and well-tolerated. PD01A triggered a rapid and long-lasting antibody response targeting the α-synuclein epitope [111].

Perspectives for treating dementia also utilize neural stem cells (NSCs) which target α-synuclein. In animals, NSC transplantation into α-synuclein transgenic mice improved cognition and motor functions [112]. The results were ascribed to the NSC-related expression of brain-derived neurotrophic factor (BDNF), which modulates dopaminergic and glutamatergic systems [112].

### 4.5. Nanotechnology-Based Approaches

Another promising option is using nano-scale carriers for drugs, whose effectiveness in central nervous system disorders has been reviewed recently [113,114,115,116,117,118]. The application of nanocarriers to treat AD has also recently been reviewed [119]. Simultaneously, great hope is also placed in nanoformulations of natural compounds, such as curcumin [120]. This attractive strategy, namely nanoencapsulation in, e.g., lipid- or polymer-based carriers, can provide superior therapeutic effects, reduced side effects, enhanced safety, targeted and precise delivery, or increased availability.

The delivery of agents to the brain is always challenging, and the additional complexity of nanocarriers increases the burden for a high level of characterization and complex manufacture. Nevertheless, several nanocarriers have been described as promising therapeutic strategies for targeting mitochondrial therapy, Aβ, or tau proteins. For example, recent research showed that using chiral D-Fe_x_Cu_y_Se nanoparticles led to the recovery of cognitive competence in vivo models. Moreover, the removal of amyloid plaques in the brain was observed after the application of tested chiral nanoparticles [121]. Another study revealed that vitamin D-binding protein-loaded PLGA nanoparticles attenuated the Aβ accumulation, neuronal loss, neuroinflammation, and cognitive dysfunction after intravenous administration in 5XFAD mice [122].

Sonawane et al. proved that protein-capped metal (PC-Fe_3_O_4_ and PC-CdS) nanoparticles inhibited tau aggregation [123]. It is worth emphasizing that the authors have also shown that protein-capped CdS nanoparticles could both disaggregate and inhibit tau [123]. In another study, neuron tau-targeting nanoparticles for curcumin delivery relieving Alzheimer’s disease symptoms were described. By using these biocompatible NPs, suppression of neuronal-like cell death and the decrease of the intracellular p-tau level was possible [124]. An alternative method using a neuronal mitochondria-targeted therapy, functionalized with dual-targeting ligands, as a novel biomimetic delivery system was presented by Han et al. [125]. Through resveratrol encapsulation, Aβ-related mitochondrial oxidative stress was mitigated, as confirmed using both in vitro and in vivo models. Additionally, the improvement of memory impairment in mice was recorded [126]. Another exciting option with great potential for application in neuroimaging is the use of nanoparticles as contrast agents for the diagnosis of AD [126].

Unfortunately, only a few nanocarrier formulations are in clinical trials (Table 4). Nanolithium (NP03), a disease-modifying nanoparticle formulation of lithium citrate in an AONYS^®^ reverse microemulsion [127], has already been tested in preclinical studies [128,129,130]. Phase 2 of the survey (ClinicalTrials.gov ID: NCT05423522) will provide information regarding the clinical safety and efficacy of NanoLithium^®^ NP03 in patients with mild-to-severe Alzheimer’s Disease (estimated completion in February 2024).

The safety, tolerability, and efficacy of intranasal nanoparticles of APH-1105, a novel alpha-secretase modulator for mild-to-moderate cognitive impairment due to AD, is currently undergoing a phase 2 study (ClinicalTrials.gov ID: NCT03806478), due for completion in December 2024. Hence, there is optimism that the number of successful trials of nanocarrier-enabled formulations for AD treatment will increase soon to realize their potential to provide new therapeutic approaches to offset the complexity and scalability difficulties of the designed nanosystems.

## 5. Conclusions

In recent years, drug discovery and development pipelines targeting dementia have been diversifying and have significantly improved. Analysis of dementia clinical trials showed conceptual supremacy for disease-modifying therapies as opposed to symptomatic-disease approaches. However, diagnostic criteria must be optimized for more rigorous randomized controlled trials, and new biomarker strategies are necessary to improve diagnostic capabilities and trial designs.

## Figures and Tables

**Table 1:** FDA-approved drugs to improve cognitive functions.

Drug	Structure	Therapeutic Effects
Donepezil	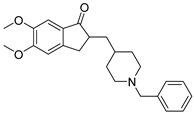	selectively and reversibly inhibits cholinesterase, improves the cognitive and behavioral signs and symptoms of AD, neuroprotective
Galantamine	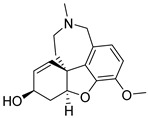	levels decrease as the disease progresses, not considered as a disease-modifying drug
Rivastigmine	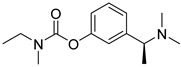	parasympathomimetic and a reversible cholinesterase inhibitor, enhancing cholinergic function
Memantine	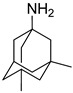	inhibits calcium influx into cells, enhances neuronal synaptic plasticity

**Table 2:** The representatives of clinical trials of drugs considered for repurposing in dementia.

Drug	Approval	Mechanism of Action	Clinical Trials ID
Nilotinib	Chronic myelogenous leukemia	Tyrosine kinase inhibitor	NCT02947893
Neflamapimod ^1^	Antiarthritic/anti-inflammatory	p38 MAP kinase alpha inhibitor	NCT03402659
Bexarotene	Anti-cancer	Retinoid X receptor agonist	NCT01782742
Liraglutide	Anti-diabetic	Glucagon-like peptide 1 agonist	NCT01469351
Rosiglitazone	Anti-diabetic	Peroxisome proliferator-activated receptor-γ agonist	NCT00265148 NCT00428090 NCT00550420
Nilvadipine	Anti-hypertensive	Calcium channel blocker	NCT02017340
Candesartan	Anti-hypertensive	Angiotensin receptor blocker	NCT02646982
Losartan	Anti-hypertensive	Angiotensin receptor blocker	EudraCT 2012–003641–15
Losartan/amlodipine	Anti-hypertensive	Angiotensin receptor blocker/Calcium channel blocker	NCT05331144
Rasagiline	Parkinson’s disease	Selective monoamine oxidase B inhibitor	NCT02359552
Roflumilast	Severe chronic obstructive pulmonary disease	Phosphodiesterase 4 inhibitor	NCT02051335 NCT02079844
Sildenafil	Erectile dysfunction, pulmonary arterial hypertension	Phosphodiesterase 5 inhibitor	NCT05039086

^1^ has been granted Fast Track status as a treatment for LBD by the FDA.

**Table 3:** The representatives of clinical trials of new small molecules targeting dementia.

Small Molecule	Mechanism of Action	Clinical Trials ID	Condition or Disease
HT-0712	Phosphodiesterase 4 inhibitor	NCT02013310	age-associated memory impairment
BPN14770	Phosphodiesterase 4 inhibitor	NCT02648672 NCT0303010 NCT02840279	safety, tolerability, and pharmacokinetic profile
PF-04447943	Phosphodiesterase 9 inhibitor	NCT00988598 NCT00930059	cognitive and behavioral symptoms of AD
BI 409306	Phosphodiesterase 9 inhibitor	NCT02392468 NCT02337907	cognitive impairment due to AD
Intepirdine	Selective 5-HT_6_ receptor antagonist	NCT02586909	long-term safety and tolerability in LBD
Nelotanserin	5-HT_2A_ inverse agonist	NCT02871427	long-term safety and tolerability in LBD
Ramelteon	Melatonin receptors MT_1_ and MT_2_ selective agonist	NCT00325728	mild-to-moderate AD
Bosutinib	Tyrosine kinases Abl/Src dual inhibitor	NCT03888222	safety, tolerability, biomarkers in LBD
Nilotinib	Abl tyrosine kinase inhibitor	NCT04002674	safety, tolerability, biomarkers in LBD
Irsenontrine	Phosphodiesterase 9 inhibitor	NCT03467152	safety, tolerability, biomarkers in LBD
Pimavanserin	5-HT_2A_ inverse agonist	NCT03325556	dementia-related psychosis
Neflamapimod	p38 mitogen-activated protein (MAP) kinase inhibitor	NCT04001517	cognitive effects in LBD

**Table 4:** Clinical status of nanocarriers.

Name	Description	Administration	Clinical Trials ID
NanoLithium^®^ NP03	Proof-of-concept study to assess safety, tolerance, and efficacy of NanoLithium^®^ NP03 in patients with mild-to-severe AD	Depositing in the gingivo-jugal groove of each cheek	NCT05423522
APH-1105	Study to assess the safety, tolerability, and efficacy of intranasal delivery of APH-1105 for the treatment of mild-to-moderate AD in adults	Intranasal	NCT03806478

## Data Availability

Not applicable.
